# Microbial identification by mass cataloging

**DOI:** 10.1186/1471-2105-7-117

**Published:** 2006-03-08

**Authors:** Zhengdong Zhang, George W Jackson, George E Fox, Richard C Willson

**Affiliations:** 1 Department of Biology and Biochemistry, University of Houston, 4800 Calhoun Avenue, Houston, TX 77204-5001, USA; 2 Department of Molecular Biophysics and Biochemistry, Yale University, New Haven, CT 06520, USA; 3 Department of Chemical Engineering, University of Houston, 4800 Calhoun Avenue, Houston, TX 77204-4004, USA; 4 BioTex, Inc., 8058 El Rio St., Houston, TX 77054, USA

## Abstract

**Background:**

The public availability of over 180,000 bacterial 16S ribosomal RNA (rRNA) sequences has facilitated microbial identification and classification using hybridization and other molecular approaches. In their usual format, such assays are based on the presence of unique subsequences in the target RNA and require a prior knowledge of what organisms are likely to be in a sample. They are thus limited in generality when analyzing an unknown sample.

Herein, we demonstrate the utility of catalogs of masses to characterize the bacterial 16S rRNA(s) in any sample. Sample nucleic acids are digested with a nuclease of known specificity and the products characterized using mass spectrometry. The resulting catalogs of masses can subsequently be compared to the masses known to occur in previously-sequenced 16S rRNAs allowing organism identification. Alternatively, if the organism is not in the existing database, it will still be possible to determine its genetic affinity relative to the known organisms.

**Results:**

Ribonuclease T_1 _and ribonuclease A digestion patterns were calculated for 1,921 complete 16S rRNAs. Oligoribonucleotides generated by RNase T_1 _of length 9 and longer produce sufficient diversity of masses to be informative. In addition, individual fragments or combinations thereof can be used to recognize the presence of specific organisms in a complex sample. In this regard, 140 strains out of 1,921 organisms (7.3%) could be identified by the presence of a unique RNase T_1_-generated oligoribonucleotide mass. Combinations of just two and three oligoribonucleotide masses allowed 54% and 72% of the specific strains to be identified, respectively. An initial algorithm for recovering likely organisms present in complex samples is also described.

**Conclusion:**

The use of catalogs of compositions (masses) of characteristic oligoribonucleotides for microbial identification appears extremely promising. RNase T_1 _is more useful than ribonuclease A in generating characteristic masses, though RNase A produces oligomers which are more readily distinguished due to the large mass difference between A and G. Identification of multiple species in mixtures is also feasible. Practical applicability of the method depends on high performance mass spectrometric determination, and/or use of methods that increase the one dalton (Da) mass difference between uracil and cytosine.

## Background

In light of growing concern over increasing microbial resistance to antibiotics, bioterrorism, and the continuous emergence and re-emergence of infectious diseases [1,2], several government agencies have identified the development of new diagnostics as a key to maintaining the health of the U.S. population and the world as a whole [3-6]. Infectious diseases account for 26 percent of total global mortality [7] and are the third leading cause of death in the United States [8,9]. In addition to microbial identification for clinical response, diagnostics are also needed for a wide range of applications including food and water safety, bioreactor analysis and sterility assurance, and environmental microbiology. In some instances, e.g. a long-term space mission, bioterror incident, or emerging disease, it may be impossible to anticipate what the problem organism(s) will be.

To this day, determinative bacteriology often relies on culture-based methods involving time-consuming isolation, cultivation, and characterization of phenotypic traits of the culture. While in a few cases a rapid identification can be made using phenotypic methods, the phylogenetic resolution of such methods is usually quite low. Characterization of cells based on morphology, staining, and metabolic traits is often not discriminatory and can take days to weeks for unambiguous identification [10,11]. Some recent advances have been made using *in vivo *phage-based detection mechanisms, however these systems require precise culturing conditions that can affect their accuracy, and increased culture time opens the window for contamination or selection [12]. Perhaps most importantly, many pathogens are extremely fastidious or even uncultivable under laboratory conditions, so that culture-based methods are not applicable. Finally, such methods are labor-intensive, not amenable to automation, and require extensive "hands-on" time and interpretation by the trained microbiologist. In the "post-genome" era, molecular methods are rapidly supplanting phenotypic characterization.

Although a variety of approaches are in use, most current microbial diagnostic research is focused on molecular methods such as comparative sequencing of PCR-amplified 16S ribosomal RNA genes (rDNA), hybridization with labeled probes or molecular beacons, phylogenetic microarrays, and reverse transcription of rRNA and amplification (RT-PCR) used in conjunction with hybridization probes or sequencing [13-15]. Methods utilizing 16S rRNA are especially common because this molecule is now widely used in the assignment of bacteria to specific taxonomic groups, and partial or full sequences are available from over 180,000 strains (as of RDP release 9.32, 72,540 sequences are of length 1200 nt or more). Methods that rely on hybridization are effective at leveraging genomic information, but they typically face the significant drawback of requiring construction of one or more probes based on *a priori *knowledge of the genus or species that needs to be detected.

Complete or partial genomic sequencing requires no such preliminary knowledge, but sequencing is time-consuming and labor-intensive. Several commercial kits are available for microbial identification using complete 16S rDNA sequencing by standard PCR (with universally conserved primers) amplification of the 16S rDNA gene followed by chain-termination sequencing [16,17]. However, read lengths are currently limited to about 500 bases, so that full coverage of the approximately 1,542-nucleotide 16S rDNA gene, requires separate sequencing of the products of three PCR reactions.

In summary, a variety of molecular methods have been developed for microbial identification. However, they are typically limited in their generality by their requirement for *a priori *knowledge of a putative characteristic sequence. Methods of full sequence determination are more general, but are labor-intensive and not readily amenable to mixtures or quantitative comparisons. Both sequencing and hybridization require a means for radioisotope- or fluorescence-labeling.

### Sequence cataloging versus sequence comparison

Before the development of modern DNA sequencing methods, oligoribonucleotide cataloging was successfully used to compare 16S rRNA sequences and determine relationships between bacteria [18]. The essence of the method was to fragment the RNA with ribonucleases, isolate the resulting small digestion products, and individually sequence each of them [19-22]. RNase T_1 _and RNase A cleave 16S rRNA specifically on the 3'-side of G residues and pyrimidine residues (C and U), respectively. Experimental fragmentation of the RNA with RNase T_1 _proved to be especially informative, reliable, and reproducible. The extent of similarity between organisms could be deduced by comparing the RNase T_1 _catalogs [23]. It was empirically found that when comparing two catalogs, identities between oligoribonucleotides of length six and longer were far more likely to be the result of sequence homology than random chance [19,24]. Thus, sequences of all smaller oligoribonucleotides were ignored in the comparisons. Like actual sequences, such catalogs are additive to a database as they are generated. In addition, any newly-determined sequence can be deconstructed to a mass catalog and included in a comparison. It is appropriate to point out that conceptually similar approaches are well accepted and have been used quite successfully in the field of proteomics – that is, partial enzymatic digestion and comparison to a database of predicted masses [25-28].

### Mass spectrometry for sequence characterization

Due to the aforementioned challenges, a number of methods for sequence characterization using mass spectrometric analysis have been proposed as alternatives to hybridization or full sequencing [29-33]. MALDI-TOF (matrix-assisted laser desorption ionization time-of-flight) mass spectrometry is a method of choice for measuring the masses of oligoribonucleotides, especially mixtures thereof. The MALDI ionization process results from absorption of laser excitation light by an aromatic organic acid and transfer of that energy to the co-crystallized analyte. Once ionized, the desorbed plume of matrix and analyte is accelerated in a vacuum through a long flight tube to a "time-of-flight" detector. The time of flight is proportional (subject to correction factors) to the square root of the mass-to-charge ratio. Because "soft-ionization" techniques such as MALDI provide a direct means of measuring one of the fundamental properties of biomolecules, they have recently been widely used for novel analytical assays. Mass spectrometric characterization of nucleic acids has been used in a variety of applications including analysis of single nucleotide polymorphisms (SNPs), and dideoxy sequencing [34,35]. When MALDI of nucleic acids first became practicable, it was quickly seen as a possible substitute for nucleic acid sequencers due to the rapidity of the separation versus capillary or gel electrophoresis. This led to most groups treating the MALDI-TOF mass spectrometer as a chain-termination (Sanger concept) sequencer. That is, nested di-deoxy chain terminated fragments were separated in the mass spectrometer and the sequence directly read from the spectrum. At present, however, the maximum read length using such an approach is ~56 nucleotides [31].

### Compositional characterization

Because of this limitation on read-length, a compromise between information loss and speed (provided by rapid mass spectrometry) has been reached. For discrimination of organisms based upon compositional information alone, either extremely accurate mass measurement of a large sequence region is necessary, or less precise measurement of multiple informative fragments is required. For example, Ecker *et al*. have employed electrospray ionization (ESI) mass spectrometry in conjunction with Fourier transform ion cyclotron resonance (FTICR) to successfully type strains of Group A streptococci and were able to distinguish them from commensal organisms [36,37]. PCR products are obtainable for a broad range of organisms using universally conserved primers and mismatch-tolerant reaction conditions. The PCR products (80–140 bp) are then analyzed for their total base compositions, i.e. A_w_C_x_G_y_T_z_. Unfortunately, the very high resolution required for unambiguous compositional assignment (± 1 ppm) of such a large molecule requires elaborate instrumentation which is out of reach for many laboratories. In contrast, the more common approach has been to generate base-specific fragments of single-stranded DNA or RNA. That is, a single-stranded sequence is cleaved after every occurrence of a particular base generating multiple fragments of smaller mass.

Herein we examine the possibility of using mass spectrometry to generate catalogs of rRNA fragment compositions (masses) as a tool for rapid microbial identification. MALDI generates singly-charged ions and is widely regarded as the mass spectrometric technology most applicable to analyzing the types of mixtures generated by nucleic acid fragmentation. In fact, the experimental feasibility of using a fragmentation strategy in combination with MALDI has already been established. Hahner *et al*. have described endoribonuclease digestion for the generation of RNase T_1 _fragments and MALDI characterization [38]. Hartmer *et al*. modified the approach and applied it to discovery of SNPs and identification of bacteria using 16S rRNA regions [39]. Von Wintzingerode *et al*. showed MALDI of base-specific fragmentation patterns of 16S rDNA amplicons to be a viable method for microbial identification and compared experimental to predicted masses for *Bordetella *species [40]. More recently Lefmann *et al*. have used base-specific cleavage to discriminate mycobacteria [41].

To our knowledge, no systematic calculations have been performed to assess the absolute identifying power or phylogenetic classifying utility of MALDI-MS when applied to RNase T_1 _or RNase A-generated fragments from a large 16S rRNA sequence dataset.

### Hypothesis and underlying issues

We hypothesized that there is sufficient discriminatory power in base-specific rRNA fragment compositions alone for identification of known and previously-unknown organisms. Moreover, the simultaneous presence of just one to three characteristic compositions may be used for unambiguous identification of many organisms, even in a complex mixture. In order to investigate the occurrence of identifying compositions in a large dataset, we performed computational, base-specific fragmentations of a large number of 16S rRNA sequences and investigated the identifying power of the fragments as distributed among the organisms. Using these results, we developed a program for simulation of MALDI-TOF mass spectrometric identification of organisms selected from the oligoribonucleotide fragment libraries.

Preliminary issues involving genetic alphabet, information degeneracy, isotopes, and instrument resolution were considered. With regard to alphabet, obvious considerations are whether DNA or RNA will be used and whether the fragments observed will be single or double-stranded. In nature, mass-modified nucleotides are occasionally present in both DNA and RNA. However, post-transcriptionally modified nucleotides in ribosomal RNA are few. Large fragments containing them are rare, and they are typically universal to large groupings of bacteria. We therefore know what specific modification-produced masses to discount [42]. Finally, they are absent from *in vitro *runoff and amplification products. Thus, it is only necessary to consider the usual four-letter alphabets (A, C, G, and T, or A, C, G, and U).

Compared to the traditional catalogs of oligoribonucleotide sequences, a catalog of fragment masses suffers from degeneracy due to the fact that multiple distinct permuted sequences generated by RNase digestion, e.g. AUCCG and UACCG, have the same composition and hence the same mass. This many-to-one relationship results in a loss of information potentially useful for microbial identification. For RNA targets, this problem is exacerbated by the small (one dalton) mass difference between C and U, raising the question of whether it is possible to distinguish RNase T_1 _oligomers differing only in the number of C's and U's.

Unequivocal determination of oligoribonucleotide composition based on mass alone is obviously dependent on the resolution and accuracy of the mass spectrometer. Koomen *et al*. have published an extensive review of the requirements for accurately determining oligonucleotide compositions from measured mass [43]. They determined that theoretically, all monoisotopic compositions of DNA up to 13 mers could be accurately assigned at 5 ppm mass accuracy or better. Operating in reflectron mode, employing proper sample preparation techniques, and including internal calibration standards, they were able to obtain 6 ppm or better accuracy on samples ranging from 5 to 13 mers. An accuracy of 6 ppm, for example, would allow assignment to within 0.0234 Da at 3900 Da, allowing discrimination of a U to C difference in composition in a 50%-purine oligoribonucleotide of length ca. 13. MALDI-TOF mass spectrometry operated in linear mode with no added internal standards is a lower resolution, lower accuracy technique, typically yielding resolution of m/Δm of 500 – 1000 and accuracy of 0.05 – 0.1% (500 – 1000 ppm) [43]. To illustrate, at an accuracy of 1000 ppm on 5000 Da (roughly the mass of a single-stranded 16-mer) a compositional assignment may be in error by 5 Da. This suggests that, though any substitution involving a purine is readily detectable, up to five substitutions of U for C or vice-versa might be indistinguishable within this hypothetical 16-mer. To address this issue experimentally, studies relying on 16S rRNA RNase T_1 _fragments will either need to be done at the highest accuracy and resolution routinely attainable or an alternative protocol will be needed wherein the mass distinction between C and U is enhanced. Alternatively, a statistical approach based on the simultaneous observation of multiple mass-fragments observed in combination may be able to unambiguously identify a microorganism even if the C/U distinction is not readily made. Oligoribonucleotides generated by RNase A will internally contain only G and A, which are readily distinguished and hence would not present a problem.

A core question that faced the original RNase T_1 _fragment cataloging approach was the oligoribonucleotide length at which a match between two organisms reflected actual sequence similarity as opposed to a chance occurrence. The number of fragments that exist for any length, n, increases as 4^n^. RNase T_1 _oligoribonucleotide digestion products, however, always contain a single G at the 3' end of the sequence and no internal Gs. Hence, the number of possible products is 3^(*n*-1)^, which for length six is 243 products (in contrast to 4^6 ^= 4096 possible 6-mers). This is significantly larger than the number of 6-mers generated by digestion of any single 16S rRNA and hence an identical match of 6-mers (or any longer fragments) between two 16S rRNAs is likely to reflect actual sequence identity rather than chance. This result was borne out by subsequent examination of complete sequences. However, the number of different mass compositions is significantly less than the number of unique sequences. It is determined by the number of 4 elements chosen n at a time with replacement:

4 choose n (with replacement)=[4+n−1n]=(n+3)!n!3!=(n+3)(n+2)(n+1)6,     (1)
 MathType@MTEF@5@5@+=feaafiart1ev1aaatCvAUfKttLearuWrP9MDH5MBPbIqV92AaeXatLxBI9gBaebbnrfifHhDYfgasaacH8akY=wiFfYdH8Gipec8Eeeu0xXdbba9frFj0=OqFfea0dXdd9vqai=hGuQ8kuc9pgc9s8qqaq=dirpe0xb9q8qiLsFr0=vr0=vr0dc8meaabaqaciaacaGaaeqabaqabeGadaaakeaacqaI0aancqqGGaaicqqGJbWycqqGObaAcqqGVbWBcqqGVbWBcqqGZbWCcqqGLbqzcqqGGaaicqqGUbGBcqqGGaaicqGGOaakcqqG3bWDcqqGPbqAcqqG0baDcqqGObaAcqqGGaaicqqGYbGCcqqGLbqzcqqGWbaCcqqGSbaBcqqGHbqycqqGJbWycqqGLbqzcqqGTbqBcqqGLbqzcqqGUbGBcqqG0baDcqGGPaqkcqGH9aqpdaWadaqaauaabeqaceaaaeaacqaI0aancqGHRaWkcqWGUbGBcqGHsislcqaIXaqmaeaacqWGUbGBaaaacaGLBbGaayzxaaGaeyypa0ZaaSaaaeaacqGGOaakcqWGUbGBcqGHRaWkcqaIZaWmcqGGPaqkcqGGHaqiaeaacqWGUbGBcqGGHaqicqaIZaWmcqGGHaqiaaGaeyypa0ZaaSaaaeaacqGGOaakcqWGUbGBcqGHRaWkcqaIZaWmcqGGPaqkcqGGOaakcqWGUbGBcqGHRaWkcqaIYaGmcqGGPaqkcqGGOaakcqWGUbGBcqGHRaWkcqaIXaqmcqGGPaqkaeaacqaI2aGnaaGaeiilaWIaaCzcaiaaxMaadaqadaqaaiabigdaXaGaayjkaiaawMcaaaaa@7A2C@

where ! denotes factorial [44]. For instance, the number of unique compositions for the complete set of possible 10 mers is 13!/(10! × 3!) or 286, which is much less than the 4^10 ^= 1,048,576 unique sequences. Because an RNase T_1 _oligoribonucleotide will always end in G, the number of possible compositions of length n can be expressed as:

(n+1)!(n−1)!2!=n(n+1)2,     (2)
 MathType@MTEF@5@5@+=feaafiart1ev1aaatCvAUfKttLearuWrP9MDH5MBPbIqV92AaeXatLxBI9gBaebbnrfifHhDYfgasaacH8akY=wiFfYdH8Gipec8Eeeu0xXdbba9frFj0=OqFfea0dXdd9vqai=hGuQ8kuc9pgc9s8qqaq=dirpe0xb9q8qiLsFr0=vr0=vr0dc8meaabaqaciaacaGaaeqabaqabeGadaaakeaadaWcaaqaaiabcIcaOiabd6gaUjabgUcaRiabigdaXiabcMcaPiabcgcaHaqaaiabcIcaOiabd6gaUjabgkHiTiabigdaXiabcMcaPiabcgcaHiabikdaYiabcgcaHaaacqGH9aqpdaWcaaqaaiabd6gaUjabcIcaOiabd6gaUjabgUcaRiabigdaXiabcMcaPaqaaiabikdaYaaacqGGSaalcaWLjaGaaCzcamaabmaabaGaeGOmaidacaGLOaGaayzkaaaaaa@46DE@

where n is still the full length of the oligoribonucleotide. RNase A fragments will end in either U or C and contain only preceding internal A's or G's. The number of possible compositions (not sequences) in this case can be expressed as:

2n!(n−1)!=2n     (3)
 MathType@MTEF@5@5@+=feaafiart1ev1aaatCvAUfKttLearuWrP9MDH5MBPbIqV92AaeXatLxBI9gBaebbnrfifHhDYfgasaacH8akY=wiFfYdH8Gipec8Eeeu0xXdbba9frFj0=OqFfea0dXdd9vqai=hGuQ8kuc9pgc9s8qqaq=dirpe0xb9q8qiLsFr0=vr0=vr0dc8meaabaqaciaacaGaaeqabaqabeGadaaakeaadaWcaaqaaiabikdaYiabd6gaUjabcgcaHaqaaiabcIcaOiabd6gaUjabgkHiTiabigdaXiabcMcaPiabcgcaHaaacqGH9aqpcqaIYaGmcqWGUbGBcaWLjaGaaCzcamaabmaabaGaeG4mamdacaGLOaGaayzkaaaaaa@3CB9@

## Results

### Mass cataloging of bacterial 16S RNA oligoribonucleotides

16S rRNA sequences from 7,322 bacterial organisms were obtained from RDP Release 7.1; 1,921 16S rRNA sequences met the selection criteria for length and quality (see Methods) and were used to generate endoribonuclease digestion products.

Table 1 gives summary statistics of complete endoribonuclease digestions of the 1,921 selected 16S rRNAs. Since RNase T_1 _cuts 16S rRNA less frequently than RNase A, a smaller number of oligoribonucleotides with a greater average length are generated. These longer oligoribonucleotides have more unique sequences and accordingly potentially unique masses. As a result, a mass catalog generated with RNase T_1 _digestion is more informative and thus more useful for bacterial identification than an RNase A catalog. Isotopic distributions of fragment masses were also calculated. In this calculation only isotopes of carbon and oxygen were considered and only peaks of more than 50% maximum relative intensity were cataloged (see Methods, pg 32). Table 1 also shows, as expected, that inclusion of isotopic elements increases the number of masses in the catalogs without changing the number of distinct oligoribonucleotide alphabetic compositions.

**Figure 1:**
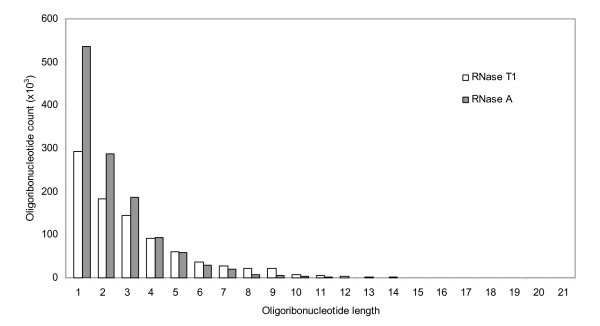
**Population distributions of 16S rRNA RNase digestion products of different lengths**. Oligoribonucleotides were generated by RNase T_1 _and RNase A digestion of 16S rRNA from 1,921 organisms. Numbers of RNase T_1 _and RNase A fragments are shown in white and gray, respectively.

**Figure 2:**
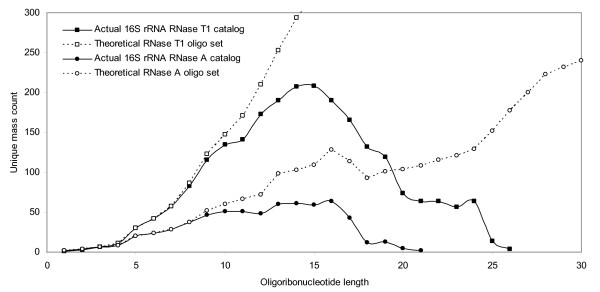
**Comparison of numbers of unique polyisotopic oligoribonucleotide masses actually occurring in digests of 16S rRNA versus possible masses**. The number of unique polyisotopic oligoribonucleotide masses in the actual RNase T_1 _and A catalogs are presented. The unique polyisotopic oligoribonucleotide masses in the theoretical sets are calculated from all possible RNase T_1 _and RNase A oligos and with consideration for the natural isotopic distribution. Only carbon and oxygen isotopes and the resultant oligoribonucleotide masses above a 50% maximum relative intensity are considered. The counts of RNase T_1_-generated oligoribonucleotide more than 26 nt long are not shown.

In terms of distinct *sequences*, the oligoribonucleotide population distributions of digestion products of different lengths (Figure 1) are similar for the two enzymes. The total number of digestion products produced is at maximum for mononucleotides (many copies of each mononucleotide are produced) and a gradual decrease is observed as length increases. Less than 1% of the oligoribonucleotides produced by RNase T_1 _contain more than 12 bases, and less than 1% of RNase A-generated oligoribonucleotides are longer than 8-mers. Although the oligoribonucleotide *mass *distributions of digestion products of different lengths are also similar for the two enzymes, they are markedly different from both the sequence population distributions (Figure 1) and the predicted number of possible compositions. Referring to Figure 2, in the mass catalogs resulting from RNase T_1 _or RNase A digestion of a typical 16S rRNA sequence, the number of distinct oligoribonucleotide masses initially increases as the oligoribonucleotide length increases, peaks around 14-mers, and then gradually decreases as the length increases further. In contrast, the overall trend of the mass counts based on theoretical composition is to increase with the oligoribonucleotide length.

At small oligonucleotide lengths, the number of predicted RNase (both A and T_1_) generated oligoribonucleotides derived from the average organism greatly exceeds the number of possible compositions for that oligoribonucleotide length, such that nearly all catalogs will be similar, and nearly complete. As can be seen from Supplementary Table 1, Sections III and IV [see Additional file 1], which take into account all isotopes above a 50% maximum intensity (see Methods), the 6-mers represent an important "cross-over" point for RNase T_1 _digestion. (Column F, sections I and II tabulate possible monoisotopic masses, while column F, sections III and IV tabulate the number of all possible distinct isotopic masses). On average, there will be less 6-mer (or larger) oligoribonucleotides observed in a 16S rRNA digest than there are possible compositions. Specifically, in a RNase T_1 _digest of an average organism's 16S rRNA, one would observe 19 6-mers while there are 42 possible isotopic masses, and the presence of particular oligoribonucleotide masses is therefore informative. For RNase A digestion, the turning point also occurs at 6-mers with 15 observed and 24 different possible isotopic masses. Longer fragments are even more distinguishing (Supplementary Table 1, column G) [see Additional file 1]. For example, the 9-mers represent another magnitude in discrimination with only one in ten predicted to occur. That is, it is an order of magnitude more likely that a matching-composition 9-mer shared between two organisms represents true underlying sequence identity than random chance. The 1-in-100 level, at which one would expect to observe less than 1% of possible compositions from the average organism, occurs for 12-mers in both the RNase A and the RNase T_1 _catalog. Detection of these and longer oligoribonucleotides is therefore highly informative.

The identifying power of the method is greatly enhanced by considering conjunctions of multiple detections, e.g. the odds of observing both a specific 11-mer and a specific 12-mer composition in a single digest. Referring to Supplemental Table 1, section III (which takes into account isotopic distribution) [see Additional file 1], for RNase T_1 _generated 11- and 12-mers, this should be approximately 0.018 × 0.007 or 1 in 7,936, which is more than the total number of organisms under consideration (1,921). This illustrates the oligonucleotide lengths which give useful discrimination even under the degeneracy that results from measuring composition (mass) instead of sequence. We have therefore identified a critical informative oligoribonucleotide length, the "1-in-10 level", reached at length 9 for both RNase T_1 _and RNase A. Shorter oligoribonucleotides are so commonly produced as to be relatively uninformative, and so only masses exceeding approximately 3,000 Da need to be considered. Furthermore, as shown in Figure 1, very few oligoribonucleotides longer than 20-mers will result from a complete digestion. Therefore, the mass-range of interest is from 3,000 to 6,000 Da. This mass-range is in stark contrast to other approaches that require unambiguous determination of the masses of much large nucleic acids (PCR products or even restriction digest products)

In accordance with earlier results of oligoribonucleotide sequence cataloging [20,22], catalogs of all the masses above these thresholds that are produced by digestion of a particular 16S rRNA will cumulatively define essentially any organism. Since the masses of interest can be determined either experimentally for an unknown organism or by analysis of a known 16S rRNA sequence, such mass catalogs can be used to determine the genetic affinity (that is, phylogenetic position based on well established 16S rRNA sequence similarity) of any unknown organism within the context of all known 16S rRNA sequences. Such mass catalogs would primarily be constructed using pure or nearly pure cultures, but they would also be useful in identifying organisms in mixtures.

### Recognition of target organisms by characteristic peaks

In many practical applications, the problem will be to determine whether a particular organism or group of organisms is present in a complex sample. In this case, rather than seeking all the masses in the spectrum that together characterize the organism of concern, the better approach may be to seek specific masses that are essentially unique to that organism or a particular phylogenetic groups of organisms. Since different molecular masses of the 16S rRNA fragments correspond to different peaks in the spectrum, every bacterium potentially has a signature peak or set of signature peaks by which it can be uniquely identified. Even if the mass-range of interest is broadened to include all 6-mers and above, as we provide motivation here, the range of ~1,800 to 6,000 Da (6- to 20-mers) is relatively narrow and well within the necessary capabilities of current instrumentation for discrimination of a large number of organisms.

RNase T_1 _and RNase A digestions of prokaryotic 16S rRNAs are not equally informative for characteristic-peak identification. For example, 140 (7.3%) of the 1,921 prokaryotes under consideration can be uniquely identified by a single RNase T_1_-generated oligoribonucleotide mass while only 27 (1.4%) can be identified by unique masses generated by the RNase A. The superiority of RNase T_1 _catalog stems from the higher specificity of RNase T_1_. Similarly, significantly more bacteria can be uniquely identified by using multiple molecular weights of the RNase T_1_-generated oligoribonucleotides together: 1,027 (53.5%) by double peaks and 1392 (72%) by triple peaks. Table 2 shows a representative sampling of the organisms that may be identified by the observation of just one to three characteristic masses generated by RNase T_1 _digestion. For example, *Camplyobacter helveticus *NCTC 12470 and *Francisella tularensis *can be uniquely distinguished by at least one RNase T_1 _oligoribonucleotide from the other 1,920 organisms sampled, and thus in theory more oligoribonucleotides are not needed to identify them. *Mycobacterium tuberculosis *does not have an RNase T_1 _oligoribonucleotide or a combination of two of them that can be used to uniquely distinguish it from other 1,920 organisms sampled. However, a unique combination of three RNase T_1 _oligoribonucleotides can be used for unambiguous identification.

### Identification of dominant bacteria in mixtures – effects of mass resolution

Although environmental samples contain numerous organisms, in many cases there are likely to be only a modest number of organisms that are dominant and metabolically-active enough to contribute a large fraction of the rRNA present. A mass spectrum of a total 16S rRNA sample isolated from such an environment will often yield a modest number of dominant peaks standing out from a large number of background peaks when digested with a ribonuclease. While direct mass spectrometric analysis of the total RNA of such a sample might yield quantitative information (at least in relative terms) about metabolic activity, the sensitivity of current MALDI instruments generally requires pre-amplification by PCR. Most commonly this has been followed by transcriptional run-off (and thereby further amplification) by phage polymerases (e.g. T7, SP6, or T3) and a return to the manipulation of RNA. Universally conserved primers would be employed to amplify the total rDNA population. In theory, so long as little bias is introduced by the PCR, the final RNA concentrations should reflect the original genomic DNA population in the mixture. This, however, may not be reflective of the metabolic activity (number of ribosomes) of the population. One solution would be to employ "linear" RNA amplification commonly used in gene-expression microarray protocols [45]. The practical considerations of DNA or RNA amplification may therefore represent an important difference in systems designed for identification (detectors) versus metabolic monitoring. Regardless of amplification steps, without additional sample preparation (see Discussion) or organism-specific PCR (recall the limitations of organism-specific hybridization probing), most observed spectra will be derived from more than one organism.

Computer simulations with different input sequences were used to test the effectiveness of mass cataloging in recovering the identity of the dominant species in such mixed samples. The assumption here is that the 16S rRNA sequences of these major organisms or their close relatives will already be in the 16S rRNA sequence database, which now exceeds 180,000 strains [46].

First, 16S rRNA sequences of four organisms – *Acidiphilium sp*. strain C-1 (*Acdp. spC1*), *Mycoplasma sturni*, strain UCMF; p170/171 ATCC 51945 (*M. sturniT*), *Methanococcus jannaschii*, rrnB gene (*Mc. janrrnB*), *Thermomonospora chromogena*, strain Agre 577 JCM 6244 (*Tmms. chrmg*) – from the filtered set of 1,921 prokaryotes were randomly selected to be mixed into a virtual sample. Two parameters, RES and WIN (see Methods), are used to simulate the real resolution limitations of a mass spectrometer and to control the number of masses selected from the entire catalog for any organism. Computer simulations were carried out with four different combinations of these parameters (Table 3). The results show that the identification of sample bacteria is best when oligoribonucleotide masses generated by RNase T_1 _digestion with RES = 1 and WIN = 3 threshold settings are used (the third simulation). This is expected given the excellent resolution of peaks in the spectrum. The unsatisfactory result in the first simulation (with RES = 1 and WIN = 1), underlines the importance of WIN, the parameter controlling how many catalog masses are selected for each observed mass. In the case of the first simulation, even though the (simulated) resolution is very good, fewer informative catalog masses occur because WIN is small, and the resulting identifications were far from ideal. The result of the second simulation shows that sample identification is at its worst when the mass spectrometry experiment has low resolution (RES = 5) and the identification program uses little information from the mass catalog (WIN = 1). The last simulation shows that when RES = 5 and WIN = 3 the identification was surprisingly good given the low resolution. This finding is significant, as it demonstrates that a well-chosen value of WIN can substantially improve any problem of low resolution.

To find out if the proposed method can correctly determine the genetic affinity (nearest relatives) of a 16S rRNA that was *not *used to make the mass catalog, we selected the 16S rRNA from *Nitrosospira sp*. Strain L115 (*Nss. spL115*). This organism was originally excluded from the data set because its length (1399 nt) is less than the criterion used (1400 nt). The three closest relatives found in the 1921 organism data set using RNase T_1 _oligoribonucleotide mass catalogs with RES = 5 and WIN = 3 were *Nss. multi5 *(92.72%), *Nss. spT7 *(92.86%), and *Nss. spD11 *(94.70%). The substantive sequence variations found between *Nss. spL115 *16S rRNA and the sequences from *Nss. multi5*, *Nss. spT7*, and *Nss. spD11 *in the multiple sequence alignment (data not shown) indicate that *Nitrosospira sp*. Strain L115 is similar to but also distinctively different from these three species of *Nitrosospira*. Together, these two results show that 16S rRNA oligoribonucleotide mass catalogs can in principle be used to determine the genetic affinity of "unknown" bacteria.

These initial results provide a method for determining the genetic affinity of organisms recovered from mixed population samples. Any organism in the data base that closely resembles an organism that is actually present in a sample will in most cases be strongly indicated. It necessarily will typically not be clear if more than one strain or possibly species of that type is actually present. Moreover, the precise identity of what is actually present will likely also not be unambiguously determined by this method alone. In many cases background information, previous experience or other tests will allow the resolution of such ambiguities for a given system.

## Discussion

For this study we implicitly assumed that the ribonuclease reactions would be taken to completion, i.e. no internal G residues would remain after RNase T_1 _digestion, and no internal pyrimidines remain after RNase A treatment. In principle, ribosomal RNA structure might protect certain regions from complete ribonuclease digestion without denaturation, but in practice incomplete digestion was never a problem with the original experimental 16S rRNA cataloging procedure. If incomplete digestion were a problem it might be readily dealt with by increased enzyme concentration, reaction times, and addition of mild denaturants and/or heat to improve the yield of the complete products.

Although approximately 1% of bases in the naturally occurring 16S rRNA are known to be post-transcriptionally modified [47] and therefore have unexpected masses, the possible presence of such modified nucleotides was not considered here, for two reasons. First, it is known that very few modifications occur in the larger RNase fragments (length 9 or more). In addition, since the fragments that contain the modifications and the modification itself are typically highly conserved, it would be straightforward to identify them and make the needed mass adjustment. Also, because *in vitro *transcription is likely be used to generate sufficient 16S rRNA to meet the sensitivity limits of contemporary MALDI instruments the actual samples will not contain the biological modifications.

Although it has already been shown that sufficient ribosomal RNA can be isolated for direct enzymatic manipulation and detection in a MALDI instrument [48], the utility of mass spectrometry in characterizing 16S rRNA would be greatly improved if one could more readily distinguish cytosine and uracil, which have very similar masses. This has typically been accomplished by first converting the mixture of 16S rRNAs in a sample to DNA templates by amplification and then using T7 runoff transcription to synthesize a larger quantity of a highly purified RNA with a nonstandard mass-modifying base [39,49]. If the mass-modification is at the 2' position, e.g. deoxyuracil instead of uracil, it increases the stability of the fragments produced and makes cleavage with RNase A monospecific. This would result in many additional and valuable unique masses of length 9 or more in the RNase A catalog. Incorporation of 2'-modified bases is accomplished through a commercially available mutant RNA polymerase (R&DNA polymerase, Epicenter) that can incorporate various non-canonical 2'-ribonucleotides composed of rNMPs (ribonucleotide-monophosphates), dNMPs (deoxyribonucleotide-monophosphates), modified 2'-NMPs or of mixed dNMP/rNMP or 2'-modified-NMP/rNMP compositions [50,51].

While this approach is certainly beneficial, the ideal base-specific fragments would have no remaining internal ribose moieties such that the final products would be stable against further non-specific cleavage or hydrolysis. In our hands, substituting more than one dNTP or 2'-modified NTP substrate for the natural rNTPs in the transcription reaction has so far resulted in insufficient yield to obtain a good MALDI-TOF signal to noise ratio or full length transcripts are not obtained, complicating the spectra. Another solution might be to employ a mutant DNA polymerase capable of incorporation of a single RNA base [52-54]. Finally, run-off transcription using amino-allyl U (aaU) is well known and has been used by us and others for incorporation of a reactive site for fluorescent labeling. Because the aminoallyl group is not a "bulky" modification (Δm = 55.08 Da), aaUTP can be completely substituted for standard UTP in a transcription reaction with full-length product still being obtained. The standard fragments in a RNase T_1_-digest of natural *E. coli *16S rRNA had nearest mass neighbors of 0.985 Da (the U/C difference in mass) while the amino-allyl modified fragments had nearest neighbors of 8.013 Da apart. That is, the mass-modification has the effect of expanding the mass spectrum. Before modification, the *E. coli *masses of interest ranged from 1938.17 – 4502.72 Da, and after aaU substitution the corresponding oligoribonucleotides had masses in the range 1993.25 – 5349.42 Da. Thus, while absolute mass did not increase greatly (which would be a detriment to resolution) nearest-neighboring peaks are distanced from one another by over 8 Da. Our simulations using amino-allyl U mass modification effectively result in resolution at three residues instead of two as is currently the case with RNase T_1 _and naturally occurring bases. Alternative strategies of making the C/U distinction such as the use 2'-methyl C and RNase A, for example, would result in the same level of alphabetic resolution.

If two cleavage reactions are used at the same time the effect is to drastically reduce the information content as each fragment produced in one reaction is further cleaved by the second. Two cleavage reactions could conceivably be performed in parallel and the results merged. We have effectively simulated that by exploring separate results for RNAse T1, RNAse A and the amino-allyl U incorporation for enhanced C/U distinction. So instead of examining the number of organisms that can be identified by using 1, 2, 3 masses etc. from a single digestion one could instead choose masses from a larger catalog of masses. The main advantage would be the availability of a larger number of discriminating masses to choose from.

**Figure 3:**
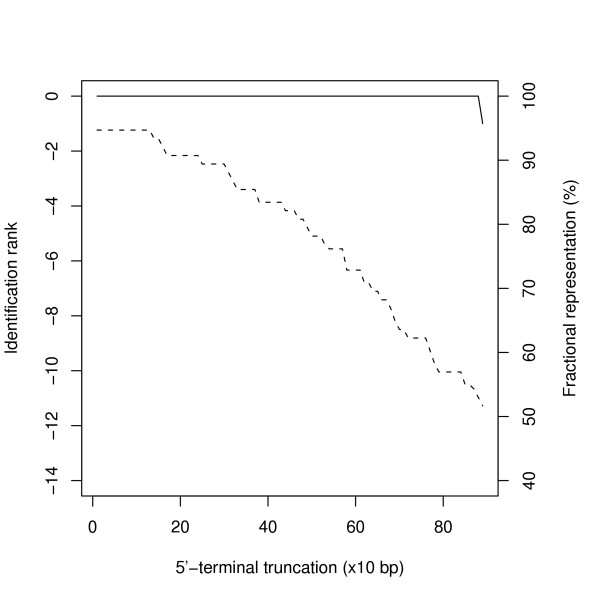
**Identification rank and representation of truncated 16S rRNA from *Nss. spL115 *with 16S sequence truncated from the 5'-end**. 16S rRNA of *Nitrosospira sp*. Strain L115 was truncated from its 5' end 10 nt a time for 89 times. Identification of each truncated 16S rRNA was simulated, with RES = 5, WIN = 3, and isotopic masses in RNase T_1 _catalog. The solid line is the identification rank, with the primary y-axis on the left. Rank 0 means one of the three *Nitrosospira *(*Nss. multi5*, *Nss. spT7*, and *Nss. spD11*) in the catalog is ranked as the highest-fractional representation bacterium in the sample. Rank -1 means one of the three *Nitrosospira *in the catalog is ranked as the second most represented bacterium in the sample, and so on. The dashed line is the fractional representation of the highest ranked *Nitrosospira *in the catalog, with the secondary y-axis on the right.

**Figure 4:**
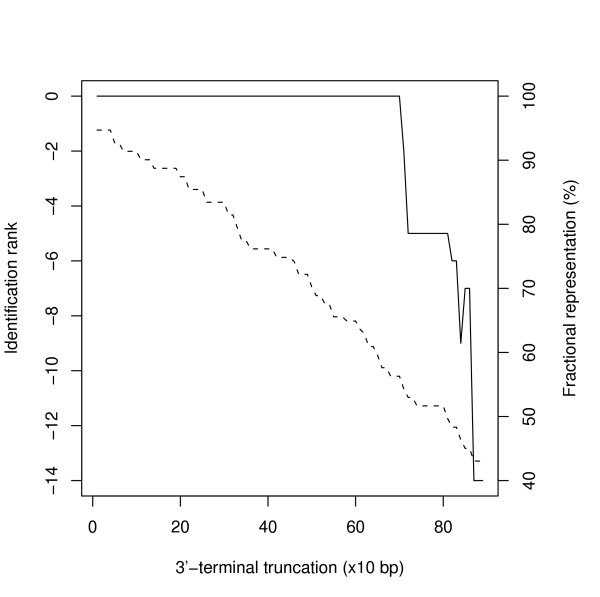
**Identification rank and representation of truncated 16S rRNA from *Nss. spL115 *with 16S sequence truncated from the 3'-end**. 16S rRNA of *Nitrosospira sp*. Strain L115 was truncated from its 3' end 10 nt a time for 89 times. As in Figure 3, identification of each truncated 16S rRNA was simulated with RES = 5, WIN = 3, and isotopic masses in RNase T_1 _catalog. Axes are identical to those in Figure 3.

Since PCR and T7 transcription may fail to amplify 16S rRNA completely, we investigated the minimum length of truncated 16S rRNA that can still be identified by this mass spectrometry approach. For this purpose, 16S rRNA of *Nitrosospira sp*. Strain L115 was truncated from its 5' end and 3' end separately, 10 nt a time up to 89 times – thus, the shortest truncated 16S rRNA is 509 nt long with 890 nt lost from either end. Identification of each truncated 16S rRNA was subsequently simulated. The results (Figure 3 and 4) show that after 880 nt were truncated from the 5'-end (519 nt left), or up to 700 nt truncated from the 3'-end (699 nt left), the proper close relatives could still be identified, although with a fractional representation (50–60%). In both cases, the representation decreased linearly as the truncation increased. The linearity of the decrease reflects the uniform distribution of characteristic oligoribonucleotides in 16S rRNAs.

Von Wintzingerode *et al*. [40] have previously argued that base-specific fragments from full-length 16S rDNA (or rRNA) would "crowd" real spectra making them too difficult to interpret. If this in fact proves to be the case, it is fortunate that several highly-conserved regions in 16S exist that will allow the 16S sequence to be divided into several amplicons, each representing approximately one third of the sequence [55]. Indeed, if PCR is used as discussed above, appropriate selection of several primers can yield full-length coverage of the 16S rDNA sequence for most organisms [36,56]. This divide-and-conquer approach would also improve the analysis, as matching masses when two catalogs are compared will be known to be generated from equivalent variable subregions of 16S rDNA (PCR amplicon lying between two universally conserved sequences). In addition, analysis of smaller subregions of 16S may reduce the critical informative length to eight, thereby increasing the information obtained. Thus, the calculations described here for whole 16S rRNA molecules can only be more favorable if variable subregions instead of whole molecules are actually used to generate mass catalogs. Given that some nucleic acid amplification strategy would likely be employed and because MALDI acquisition is so rapid, there would be little time penalty associated with dividing the experiment into the analysis of fragments derived from several different PCR amplicons. Clearly it should be possible and beneficial to identify the most "mass-distinctive" sequence regions of 16S and we have developed preliminary tools for doing so. We also believe it is equally important to identify sequence regions which are "universally" amplifiable, so that broad organism coverage can be attained in the same assay. Exactly how to deal with the organism-multiplexed spectra that might result is an experimental issue outside the scope of this paper, however, the problem of mixtures in mass spectrometry has been approached [57,58] and protocols are also feasible for "sorting by dilution" as we discuss below.

The effort described above to understand the potential utility of a mass cataloging approach in the analysis of a mixed sample with multiple unknown dominant species suggests that the approach is in fact very promising. At first sight, one might be disappointed by the modest number of strains that can be recognized by a single peak. However, it is imperative to understand that 16S rRNA has its greatest resolving power at the genus and higher levels of genetic relation. Comparison of 16S rRNA sequences is definitely not the method of choice for distinguishing virulent and avirulent strains, etc. It is, however, the primary method to determine the genetic affinity of an unknown organism. It is always effective to the genus level and in most cases can distinguish species. However, when multiple strains of essentially the same species are examined there frequently is little or no variation in the underlying RNA sequence itself [59,60]. Hence, mass catalogs of two strains of the same species will frequently be identical and no single mass or set of masses would distinguish them. As more and more sequences are added to the databases there will inevitably be multiple strains of essentially every species. The solution to this problem will be to preprocess the data in order to identify all the strains in the database whose 16S rRNA sequences are effectively identical (e.g. within fifteen sequence changes in a two-way comparison) as a species cluster. The mass approach would then be considered successful when assignment to one of these species clusters is possible. Since no such preprocessing was used here and many species clusters were likely present, the results given are essentially a worst-case scenario.

The assumption here is also that most observed spectra will be due to a complex organism mixture. Approaches such as "dilution-to-extinction", however, have been described in which replicate spectra are usually due to a single organism in the mixture [36]. The essence of the method is to dilute the total genomic DNA prior to input in the initial PCR step such that, on average, each PCR has a single molecular template. Replicate, "organism-pure" spectra are then observed, and relative organism abundances may be reported. In consideration of the complete fragment pattern of, say, 6-mers and above, a wide variety of statistical techniques may be appropriate for analysis of the acquired spectra including principle component analysis, pattern recognition algorithms, correlations, convolutions, or transforms, and we have promising initial results on using novel spectral comparison metrics for this purpose. Conceptually, the approach described here relies on comparing observed mass-spectra to pre-calculated virtual spectra much resembling barcodes. Because time-of-flight spectra are inherently digitized, pre-processing and comparison to virtual mass spectra would be automated. It may become convenient to give the calculated, barcode-like spectra some finite and practical peak-width. Calculated fragment patterns could then be correlated through the observed spectra and a confidence index for the presence of each organism could be derived. In such an approach, closely related organisms are likely to give similar correlation coefficients or confidence indices with calculated spectra, so an iterative return to the mass spectrum or a closer inspection of a particular mass range may be warranted.

## Conclusion

A computational assessment of the feasibility of using mass spectrometry of fragmented rRNAs to determine the genetic affinity of unknown bacteria in monocultures and mixtures was undertaken. Mass catalogs of RNase-generated fragments of 16S rRNA were shown to be extremely promising for this purpose. When full-length sequences digested with RNase T_1 _were considered, it was found that essentially all fragments of length ten or more will be informative. The approach would be able to take advantage of the natural amplification associated with rRNA and will be ideal in situations such as long-duration space flight where reliance on sequencing would be unrealistic. Although not yet fully explored, it appears that the approach will be effective with mixtures as well. It is also clear that enhancements such as improvement in the distinction between cytosine and uracil and the possible use of subregions of the RNA may further improve performance.

## Methods

### 16S rRNA sequence dataset selection

16S rRNA sequences from 7,322 prokaryotic organisms were obtained from RDP Release 7.1 [46]. These sequences are of varying quality – some were fully determined in terms of both the length and every position of the sequence while others are either partially determined and/or contain undetermined positions. Any sequence having less than 1,400 nucleotides (full length 16S is typically 1,542 nucleotides) or undetermined nucleotides was filtered out resulting in a set of 1,921 high-quality 16S rRNA sequences.

### *In silico *endoribonuclease digestion

The sequences of the oligoribonucleotides that would be produced by RNase T_1 _or RNase A digestion of the 1,921 rRNA sequences were generated by a computer program. During the *in silico *digestion process, for each 16S rRNA the set of oligoribonucleotides produced was tabulated and analyzed. The length, frequency of occurrence, and isotopic mass of each fragment was cataloged. To circumvent the problem caused by similar molecular masses of uracil and cytosine, it was sometimes assumed that 5-(3-aminoallyl)-uracil was used instead (Δm = 55.08 Da). Post-transcriptionally modified nucleotides were not considered. Due to their rarity, oligoribonucleotides generated from the 5' and 3' ends of 16S rRNAs were excluded from further consideration. Only the relative abundances of carbon and oxygen isotopes were used to calculate the isotopic mass distribution of each oligoribonucleotide. This simplification gives a satisfactory approximation with the advantage of significantly reducing computational complexity and run times. Only the resulting isotopic masses of more than 50% of the maximum signal intensity were retained. In general, this resulted in the monoisotopic mass and at least one to two "daughter" masses being retained in the catalog. Two auxiliary data structures, which map each 16S rRNA to the set of oligoribonucleotide masses that it can generate and each oligoribonucleotide mass to the set of 16S rRNAs that it can be generated from, respectively, were also derived from the oligoribonucleotide catalog to facilitate downstream analysis.

### Determination of unique singlet, doublet, and triplet mass identifiers

To tabulate the minimum number of characteristic masses necessary to distinguish an organism (see Table 2), masses that were found exclusively in a single organism were recorded. For signature doublets and triplets, exhaustive intersections were taken of the sets of all of the possible organisms that might have contributed each mass. As a simplified example, consider that after digestion of 20 sequences (organisms) only 7 masses, m1 through m7 were present in the RNase T_1_-generated library. For each of the 7 masses in the library, a set of organisms, set A through set G contributing that RNase T_1 _fragment was recorded. To identify a unique doublet exclusively "owned" by an organism, all pairwise intersections of the organism sets, AnB, AnC, ..., BnC, BnD, ... FnG, was taken. These two-fragment organism lists were, naturally, shorter than the lists of organisms containing single fragments, reflecting the greater identifying specificity of mass-doublets. When a pairwise intersection contained only one organism, the mass-doublet was recorded as a unique identifier. Similarly, unique mass-triplets were identified by taking all of the triple intersections AnBnC, AnBnD, ..., EnFnG to produce reduced lists of all organisms containing all three of those fragments. Once an intersection yielded a set containing only one organism, the corresponding masses and the organism were recorded. It is noteworthy that there may exist other unique doublets or triplets for a given organism, but only the first ones encountered (working in increasing mass order) were recorded in the present work, tending to favor the lower-mass fragments. The trivial cases of a singlet unique to a particular organism, paired with any other masses to form a unique doublet or triplet, were ignored. The algorithm can also identify quartets and higher order intersections, albeit at a rapidly increasing computational cost.

### Simulated Identification of sample bacteria

To simulate the identification of sample bacteria by 16S rRNA fragment mass spectrometry, several 16S rRNA sequences were selected to make a virtual sample. The sample 16S rRNAs were then treated *in silico *with RNase T_1 _to generate a pool of different oligoribonucleotides. (RNase A was not used for sample bacteria identification.) This pool of oligoribonucleotide sequences is in turn mapped into a collection of isotopic oligoribonucleotide masses. Conversely, each individual oligoribonucleotide mass may be attributed to a number of bacteria.

To simulate the resolution limitation of mass spectrometry in reality, on the simulated spectrometry chart mass peaks that are closer to each other than a preset resolution threshold (RES, ~1–5 Da) are merged into one peak that centers at the averaged masses of componential peaks. After merging, to select nearby catalog masses for each sample mass a second window threshold (WIN, ~1–3 Da) is used. If the absolute difference between the sample mass and a catalog mass is less than this threshold, then that catalog mass is selected. The fraction representation of bacterium *i *in the selected catalog mass distribution is Fi=NichartNicatalog⁡
 MathType@MTEF@5@5@+=feaafiart1ev1aaatCvAUfKttLearuWrP9MDH5MBPbIqV92AaeXatLxBI9gBaebbnrfifHhDYfgasaacH8akY=wiFfYdH8Gipec8Eeeu0xXdbba9frFj0=OqFfea0dXdd9vqai=hGuQ8kuc9pgc9s8qqaq=dirpe0xb9q8qiLsFr0=vr0=vr0dc8meaabaqaciaacaGaaeqabaqabeGadaaakeaacqWGgbGrdaWgaaWcbaGaemyAaKgabeaakiabg2da9maalaaabaGaemOta40aa0baaSqaaiabdMgaPbqaaiabdogaJjabdIgaOjabdggaHjabdkhaYjabdsha0baaaOqaaiabd6eaonaaDaaaleaacqWGPbqAaeaacqWGJbWycqWGHbqycqWG0baDcqWGHbqycyGGSbaBcqGGVbWBcqGGNbWzaaaaaaaa@4611@, where Nichart
 MathType@MTEF@5@5@+=feaafiart1ev1aaatCvAUfKttLearuWrP9MDH5MBPbIqV92AaeXatLxBI9gBaebbnrfifHhDYfgasaacH8akY=wiFfYdH8Gipec8Eeeu0xXdbba9frFj0=OqFfea0dXdd9vqai=hGuQ8kuc9pgc9s8qqaq=dirpe0xb9q8qiLsFr0=vr0=vr0dc8meaabaqaciaacaGaaeqabaqabeGadaaakeaacqWGobGtdaqhaaWcbaGaemyAaKgabaGaem4yamMaemiAaGMaemyyaeMaemOCaiNaemiDaqhaaaaa@362A@ is the number of peaks on the mass spectrometry chart that can be attributed to the bacterium *i *and Nicatalog
 MathType@MTEF@5@5@+=feaafiart1ev1aaatCvAUfKttLearuWrP9MDH5MBPbIqV92AaeXatLxBI9gBaebbnrfifHhDYfgasaacH8akY=wiFfYdH8Gipec8Eeeu0xXdbba9frFj0=OqFfea0dXdd9vqai=hGuQ8kuc9pgc9s8qqaq=dirpe0xb9q8qiLsFr0=vr0=vr0dc8meaabaqaciaacaGaaeqabaqabeGadaaakeaacqWGobGtdaqhaaWcbaGaemyAaKgabaGaem4yamMaemyyaeMaemiDaqNaemyyaegcbiGae8hBaWMae83Ba8Mae83zaCgaaaaa@38CD@ is the number of peaks that bacterium *i *can generate.

The sample agent identification program gives a list of all the bacteria and their corresponding fractional representations in the sample spectrum. In addition, the program allowed user-defined adjustment of the instrumental (MALDI-TOF) resolution to determine the discriminatory power of the approach using spectra ranging from idealized, atomic peaks to spectra having finite, practical peak widths.

## Authors' contributions

ZZ wrote the programs for generating the results described here, carried out initial and new analyses of the data, created the initial draft manuscript, and revised the manuscript prior to its submission. GWJ conceived of the approach for quickly determining the unique mass-doublets and triplets, collected relevant literature, and expanded and revised the manuscript. GEF oversaw the design of the programs, introduced the idea of critical length, and prepared the final manuscript. RCW conceived the study, and participated in its design and coordination and helped to draft the manuscript. All authors read and approved the final manuscript.

## Supplementary Material

Additional File 1 **"Statistics of the 16S rRNA Oligoribonucleotide Catalogs"**, Includes all data for RNase digestions of sequences under consideration including: Average Number of Oligo of a given length per 16S, and Average Number of Oligo per 16S per Number of Possible Masses. These data are presented for both RNase A and RNase T_1 _digestion with and without isotopic distribution taken into consideration (see text). Click here for file

## Figures and Tables

**Table 1:** Comparison between RNase T_1 _and RNase A 16S rRNA catalogs. See Supplementary Table for more details.

Enzyme	Attributes of the oligoribonucleotide catalog^†^					
	
	Length Range	Total Oligos	Distinct Oligos	Distinct Masses	Avg. Oligo Seqs per 16S	Avg. Masses per 16S
*Without isotopes*:						
RNase T_1_	1 – 54	898,494	8,601	858	128	77
RNase A	1 – 21	1,225,481	1,994	227	81	50
						
*With isotopes (see Methods)*:						
RNase T_1_	1 – 54	898,494	8,601	2,404	128	159
RNase A	1 – 21	1,225,481	1,994	644	81	88

† The columns are:

Length Range: the minimum and maximum length of oligoribonucleotides in catalogs.

Total Oligos: the number of all oligoribonucleotides generated by complete RNase digestion.

Distinct Oligos: the number of different oligoribonucleotide sequences in the catalogs.

Distinct Masses: the number of different oligoribonucleotide masses in the catalogs.

Avg. Oligos per 16S: the average number of different oligoribonucleotide sequences generated by every 16S rRNA RNase digestion.

Avg. Masses per 16S: the average number of different oligoribonucleotide masses generated by every 16S rRNA RNase digestion.

**Table 2:** Examples of organisms that can be identified by the observation of one, two, or three RNase T_1_-generated 16S rRNA masses.

	I	II	III
*Camplyobacter helveticus *NCTC 12470	**Y**	-	-
*Chlamydophila pneumoniae*, str. TW-183	N	**Y**	-
*Chlamydia muridarum*	N	**Y**	-
*Chlamydia suis*, str. S45	N	**Y**	-
*Chlamydia trachomatis*, str. HAR-13	N	**Y**	-
*Clostridium botulinum*			
str. KYT0-F	N	**Y**	-
str. NCA 213 B ATCC 7949	N	**Y**	-
str. 2023 ATCC 17851	N	**Y**	-
str. Eklund 202 F ATCC 23387	N	N	**Y**
str. Eklund 17B ATCC 25765	N	**Y**	-
Langeland NCTC 10281	N	**Y**	-
NCTC 7272	N	**Y**	-
NCTC 7273	N	N	**Y**
str. 468 toxin type C	N	**Y**	-
*Escherichia coli*	N	**Y**	-
*Francisella tularensis*	**Y**	-	-
*Mycobacterium tuberculosis*	N	N	**Y**
*Mycobacterium tuberculosis*, str. H37/Rv	N	**Y**	-
*Staphylococcus sciuri *ATCC 20345	N	**Y**	-
*Vibrio chelerae*, str. 1094 ATCC 14035	N	**Y**	-
*Vibrio proteolyticus*	N	**Y**	-

A 'Y' in column I indicates that a uniquely identifying RNase T_1 _oligoribonucleotide composition was found that was not present in any of the other 1,920 organisms sampled. A 'Y' in Column II or III indicates that two or three masses respectively were found that when taken together are uniquely identifying. An "N" indicates that the calculation did not yield a one, two, or three peak signature. "-" indicates that further determinations were not necessary.

**Table 3:** Simulation results for sample identification with four different RES and WIN settings.^†^

		RES (sample mass resolution threshold, daltons)			
			
		1		5	
WIN (catalog mass selection threshold, daltons)	1	*Gab.diaztr*	89.29%	* Mc.janrrnB	74.83%
		*Rpl.globi2*	90.91%	*Adm.metha2*	75.00%
		* Acdp.spC1	91.55%	*Gab.diaztr*	76.43%
		*Mc.janrrnA*	95.10%	*Bif.lactis*	76.81%
		* Mc.janrrnB	95.80%	*Rpl.globi2*	77.27%
	3	*Mc.janrrnA*	97.90%	*Rpl.globi2*	97.73%
		* Acdp.spC1	100.00%	*Mc.janrrnA*	97.90%
		* M.sturniT	100.00%	* Acdp.spC1	100.00%
		* Mc.janrrnB	100.00%	* Mc.janrrnB	100.00%
		* Tmms.chrmg	100.00%	* Tmms.chrmg	100.00%

† Bacteria in the virtual test sample are *Acdp. spC1*, *M. sturniT*, *Mc. janrrnB*, and *Tmms. chrmg*. Only bacteria identified to be in the sample with five highest fractional representations are listed.

* True positive identification.
