# Supplementary material for: Microbial identification by mass cataloging
Source: BMC Bioinformatics. 2006 Mar 8;7:117. doi: 10.1186/1471-2105-7-117 (PMC1488874; doi:10.1186/1471-2105-7-117)
Supplement: Additional File 1 — "Statistics of the 16S rRNA Oligoribonucleotide Catalogs", Includes all data for RNase digestions of sequences under consideration including: Average Number of Oligo of a given length per 16S, and Average Number of Oligo per 16S per Number of Possible Masses. These data are presented for both RNase A and RNase T1 digestion with and without isotopic distribution taken into consideration (see text). [file 1471-2105-7-117-S1.doc]

| **Supplementary Table 1: Statistics of the 16S rRNA Oligoribonucleotide Catalogs †** | | | | | | |
| --- | --- | --- | --- | --- | --- | --- |
|  |  |  |  |  |  |  |
| A | B | C | D | E | F | G |
| OligoLen, n | NumTotalOligo | NumUniqOligo  Sequence | NumUniq OligoMasses  Observed in All Catalogs | AverageNum  Oligo per 16S | NumPossible n-mer  Masses | Average n-mer per 16S per possible number of masses (E/F) |
|  |  |  |  |  |  |  |
| *I. RNase T1 digestion, without U modification, without isotopes* | | | | |  |  |
|  |  |  |  |  |  |  |
| 1 | 291,385 | 1 | 1 | 151.7 | 1 | 151.700 |
| 2 | 181,894 | 3 | 3 | 94.7 | 3 | 31.567 |
| 3 | 143,140 | 9 | 6 | 74.5 | 6 | 12.417 |
| 4 | 90,546 | 27 | 10 | 47.1 | 10 | 4.710 |
| 5 | 59,386 | 81 | 15 | 30.9 | 15 | 2.060 |
| 6 | 36,504 | 243 | 21 | 19 | 21 | **0.905** |
| 7 | 27,210 | 685 | 28 | 14.2 | 28 | 0.507 |
| 8 | 22,117 | 1,211 | 35 | 11.5 | 36 | 0.319 |
| 9 | 22,476 | 1,495 | 41 | 11.7 | 45 | 0.260 |
| 10 | 7,802 | 1,219 | 50 | 4.1 | 55 | **0.075** |
| 11 | 5,911 | 966 | 55 | 3.1 | 66 | 0.047 |
| 12 | 2,760 | 619 | 59 | 1.4 | 78 | 0.018 |
| 13 | 2,023 | 493 | 59 | 1.1 | 91 | 0.012 |
| 14 | 2,564 | 449 | 66 | 1.3 | 105 | 0.012 |
| 15 | 628 | 301 | 65 | 0.3 | 120 | **0.003** |
| 16 | 625 | 242 | 57 | 0.3 | 136 | 0.002 |
| 17 | 650 | 198 | 55 | 0.3 | 153 | 0.002 |
| 18 | 169 | 80 | 51 | 0.1 | 171 | 0.001 |
| 19 | 246 | 89 | 45 | 0.1 | 190 | 0.001 |
| 20 | 215 | 47 | 27 | 0.1 | 210 | 0.000 |
| 21 | 83 | 33 | 23 | 0 | 231 | 0.000 |
| 22 | 43 | 26 | 23 | 0 | 253 | 0.000 |
| 23 | 36 | 29 | 21 | 0 | 276 | 0.000 |
| 24 | 57 | 33 | 23 | 0 | 300 | 0.000 |
| 25 | 6 | 5 | 4 | 0 | 325 | 0.000 |
| 26 | 1 | 1 | 1 | 0 | 351 | 0.000 |
| 28 | 3 | 3 | 3 | 0 | 406 | 0.000 |
| 29 | 3 | 2 | 2 | 0 | 435 | 0.000 |
| 30 | 4 | 4 | 3 | 0 | 465 | 0.000 |
| 31 | 4 | 4 | 3 | 0 | 496 | 0.000 |
| 34 | 1 | 1 | 1 | 0 | 595 | 0.000 |
| 39 | 1 | 1 | 1 | 0 | 780 | 0.000 |
| 54 | 1 | 1 | 1 | 0 | 1,485 | 0.000 |
|  |  |  |  |  |  |  |
| Total 898,494 oligos are generated by RNase T1 cut. | | | |  |  |  |
| Of which there are 8,601 distinct oligo sequences | | | |  |  |  |
| Of which there are 858 distinct molecular masses | | | |  |  |  |
| Avg num oligo seqs/16S is 127.65 | | |  |  |  |  |
| Avg num masses/16S is 76.62 | | |  |  |  |  |
|  |  |  |  |  |  |  |
|  |  |  |  |  |  |  |
| *II. RNase A digestion, without U modification, without isotopes* | | | | |  |  |
|  |  |  |  |  |  |  |
| 1 | 533,564 | 2 | 2 | 277.8 | 2 | 138.900 |
| 2 | 285,798 | 4 | 4 | 148.8 | 4 | 37.200 |
| 3 | 186,181 | 8 | 6 | 96.9 | 6 | 16.150 |
| 4 | 92,611 | 16 | 8 | 48.2 | 8 | 6.025 |
| 5 | 58,543 | 32 | 10 | 30.5 | 10 | 3.050 |
| 6 | 28,810 | 64 | 12 | 15 | 12 | 1.250 |
| 7 | 19,675 | 128 | 14 | 10.2 | 14 | **0.729** |
| 8 | 8,147 | 243 | 16 | 4.2 | 16 | 0.263 |
| 9 | 5,201 | 307 | 16 | 2.7 | 18 | 0.150 |
| 10 | 3,896 | 344 | 17 | 2 | 20 | **0.100** |
| 11 | 1,271 | 261 | 17 | 0.7 | 22 | 0.032 |
| 12 | 681 | 204 | 16 | 0.4 | 24 | 0.017 |
| 13 | 678 | 203 | 16 | 0.4 | 26 | 0.015 |
| 14 | 141 | 62 | 16 | 0.1 | 28 | **0.004** |
| 15 | 135 | 50 | 16 | 0.1 | 30 | 0.003 |
| 16 | 82 | 30 | 16 | 0 | 32 | 0.000 |
| 17 | 47 | 20 | 13 | 0 | 34 | 0.000 |
| 18 | 10 | 7 | 4 | 0 | 36 | 0.000 |
| 19 | 7 | 6 | 5 | 0 | 38 | 0.000 |
| 20 | 2 | 2 | 2 | 0 | 40 | 0.000 |
| 21 | 1 | 1 | 1 | 0 | 42 | 0.000 |
|  |  |  |  |  |  |  |
| Total 1,225,481 oligos are generated by RNase A cut. | | | |  |  |  |
| Of which there are 1,994 distinct oligo sequences | | | |  |  |  |
| Of which there are 227 distinct molecular masses | | | |  |  |  |
| Avg num oligo seqs/16S is 81.44 | | |  |  |  |  |
| Avg num masses/16S is 50.36 | | |  |  |  |  |
|  |  |  |  |  |  |  |
|  |  |  |  |  |  |  |
| *III. RNase T1 digestion, with U modification, with isotopes* | | | | |  |  |
|  |  |  |  |  |  |  |
| 1 | 291,385 | 1 | 1 | 151.7 | 1 | 151.700 |
| 2 | 181,894 | 3 | 3 | 94.7 | 3 | 31.567 |
| 3 | 143,140 | 9 | 6 | 74.5 | 6 | 12.417 |
| 4 | 90,546 | 27 | 11 | 47.1 | 11 | 4.282 |
| 5 | 59,386 | 81 | 30 | 30.9 | 30 | 1.030 |
| 6 | 36,504 | 243 | 42 | 19 | 42 | **0.452** |
| 7 | 27,210 | 685 | 57 | 14.2 | 57 | 0.249 |
| 8 | 22,117 | 1,211 | 83 | 11.5 | 86 | 0.134 |
| 9 | 22,476 | 1,495 | 115 | 11.7 | 123 | **0.095** |
| 10 | 7,802 | 1,219 | 135 | 4.1 | 147 | 0.028 |
| 11 | 5,911 | 966 | 141 | 3.1 | 171 | 0.018 |
| 12 | 2,760 | 619 | 173 | 1.4 | 210 | **0.007** |
| 13 | 2,023 | 493 | 190 | 1.1 | 253 | 0.004 |
| 14 | 2,564 | 449 | 207 | 1.3 | 294 | 0.004 |
| 15 | 628 | 301 | 208 | 0.3 | 322 | 0.001 |
| 16 | 625 | 242 | 190 | 0.3 | - | - |
| 17 | 650 | 198 | 165 | 0.3 | - | - |
| 18 | 169 | 80 | 132 | 0.1 | - | - |
| 19 | 246 | 89 | 119 | 0.1 | - | - |
| 20 | 215 | 47 | 74 | 0.1 | - | - |
| 21 | 83 | 33 | 64 | 0 | - | - |
| 22 | 43 | 26 | 64 | 0 | - | - |
| 23 | 36 | 29 | 56 | 0 | - | - |
| 24 | 57 | 33 | 64 | 0 | - | - |
| 25 | 6 | 5 | 14 | 0 | - | - |
| 26 | 1 | 1 | 4 | 0 | - | - |
| 28 | 3 | 3 | 12 | 0 | - | - |
| 29 | 3 | 2 | 8 | 0 | - | - |
| 30 | 4 | 4 | 12 | 0 | - | - |
| 31 | 4 | 4 | 12 | 0 | - | - |
| 34 | 1 | 1 | 4 | 0 | - | - |
| 39 | 1 | 1 | 4 | 0 | - | - |
| 54 | 1 | 1 | 4 | 0 | - | - |
|  |  |  |  |  |  |  |
| Total 898,494 oligos are generated by RNase T1 cut. | | | |  |  |  |
| Of which there are 8,601 distinct oligo sequences | | | |  |  |  |
| Of which there are 2,404 distinct molecular masses | | | |  |  |  |
| Avg num oligo seqs/16S is 127.65 | | |  |  |  |  |
| Avg num masses/16S is 159.34 | | |  |  |  |  |
|  |  |  |  |  |  |  |
|  |  |  |  |  |  |  |
| *IV. RNase A digestion, with U modification, with isotopes* | | | | |  |  |
|  |  |  |  |  |  |  |
| 1 | 533,564 | 2 | 2 | 277.8 | 2 | 138.900 |
| 2 | 285,798 | 4 | 4 | 148.8 | 4 | 37.200 |
| 3 | 186,181 | 8 | 6 | 96.9 | 6 | 16.150 |
| 4 | 92,611 | 16 | 8 | 48.2 | 8 | 6.025 |
| 5 | 58,543 | 32 | 20 | 30.5 | 20 | 1.525 |
| 6 | 28,810 | 64 | 24 | 15 | 24 | **0.625** |
| 7 | 19,675 | 128 | 28 | 10.2 | 28 | 0.364 |
| 8 | 8,147 | 243 | 37 | 4.2 | 37 | 0.114 |
| 9 | 5,201 | 307 | 46 | 2.7 | 52 | **0.052** |
| 10 | 3,896 | 344 | 51 | 2 | 60 | 0.033 |
| 11 | 1,271 | 261 | 51 | 0.7 | 66 | 0.011 |
| 12 | 681 | 204 | 48 | 0.4 | 72 | **0.006** |
| 13 | 678 | 203 | 60 | 0.4 | 98 | 0.004 |
| 14 | 141 | 62 | 61 | 0.1 | 103 | 0.001 |
| 15 | 135 | 50 | 59 | 0.1 | 109 | 0.001 |
| 16 | 82 | 30 | 64 | 0 | 128 | 0.000 |
| 17 | 47 | 20 | 43 | 0 | 114 | 0.000 |
| 18 | 10 | 7 | 12 | 0 | 93 | 0.000 |
| 19 | 7 | 6 | 13 | 0 | 101 | 0.000 |
| 20 | 2 | 2 | 5 | 0 | 104 | 0.000 |
| 21 | 1 | 1 | 2 | 0 | 108 | 0.000 |
|  |  |  |  |  |  |  |
| Total 1,225,481 oligos are generated by RNase A cut. | | | |  |  |  |
| Of which there are 1,994 distinct oligo sequences | | | |  |  |  |
| Of which there are 644 distinct molecular masses | | | |  |  |  |
| Avg num oligo seqs/16S is 81.44 | | |  |  |  |  |
| Avg num masses/16S is 88.39 | | |  |  |  |  |
|  |  |  |  |  |  |  |
| † The columns are: | |  |  |  |  |  |
| A: oligo length | |  |  |  |  |  |
| B: number of total oligos generated by RNase digestion | | | | |  |  |
| C: number of oligos with unique sequences | | |  |  |  |  |
| D: number of oligos with unique masses | | |  |  |  |  |
| E: average number of oligos generated from each 16S rRNA | | | | |  |  |
| F: number of n-mers masses. In section III, this number is only available for oligos up to 15 nt long. | | | | | | |
| G: ratio of number of n-mer masses to number of oligos with unique masses (column E / column F) | | | | | | |
